# Residual Corticosteroid Production in Autoimmune Addison Disease

**DOI:** 10.1210/clinem/dgaa256

**Published:** 2020-05-11

**Authors:** Åse Bjorvatn Sævik, Anna-Karin Åkerman, Paal Methlie, Marcus Quinkler, Anders Palmstrøm Jørgensen, Charlotte Höybye, Aleksandra J Debowska, Bjørn Gunnar Nedrebø, Anne Lise Dahle, Siri Carlsen, Aneta Tomkowicz, Stina Therese Sollid, Ingrid Nermoen, Kaja Grønning, Per Dahlqvist, Guri Grimnes, Jakob Skov, Trine Finnes, Susanna F Valland, Jeanette Wahlberg, Synnøve Emblem Holte, Katerina Simunkova, Olle Kämpe, Eystein Sverre Husebye, Sophie Bensing, Marianne øksnes

**Affiliations:** 1 Department of Clinical Science, University of Bergen, Norway; 2 K.G. Jebsen Center for Autoimmune Disorders, University of Bergen, Bergen, Norway; 3 Department of Medicine, Örebro University Hospital, Örebro, Sweden; 4 Department of Molecular Medicine and Surgery, Karolinska Institutet, Stockholm, Sweden; 5 Department of Medicine, Haukeland University Hospital, Bergen, Norway; 6 Endocrinology in Charlottenburg, Berlin, Germany; 7 Department of Endocrinology, Oslo University Hospital, Oslo, Norway; 8 Department of Endocrinology, Metabolism and Diabetes, Karolinska University Hospital, Stockholm, Sweden; 9 Department of Medicine, Vestfold Hospital Trust, Tønsberg, Norway; 10 Department of Internal Medicine, Haugesund Hospital, Haugesund, Norway; 11 Department of Endocrinology, Stavanger University Hospital, Stavanger, Norway; 12 Department of Medicine, Sørlandet Hospital, Kristiansand, Norway; 13 Department of Medicine, Drammen Hospital, Vestre Viken Health Trust, Drammen, Norway; 14 Department of Endocrinology, Akershus University Hospital, Lørenskog, Norway; 15 Department of Public Health and Clinical Medicine, Umeå University, Umeå, Sweden; 16 Division of Internal Medicine, University Hospital of North Norway, Tromsø, Norway; 17 Tromsø Endocrine Research Group, Department of Clinical Medicine, UiT the Arctic University of Norway, Tromsø, Norway; 18 Section of Endocrinology, Innlandet Hospital Trust, Hamar, Norway; 19 Department of Endocrinology and Department of Health, Medicine and Caring Sciences, Linköping University, Linköping, Sweden; 20 Department of Medicine, Sørlandet Hospital, Arendal, Norway; 21 Department of Medicine (Solna), Karolinska University Hospital, Karolinska Institutet, Stockholm, Sweden

**Keywords:** Adrenal failure, adrenal steroids, Autoimmune Addison disease, cortisol, primary adrenal insufficiency, residual function

## Abstract

**Context:**

Contrary to current dogma, growing evidence suggests that some patients with autoimmune Addison disease (AAD) produce corticosteroids even years after diagnosis.

**Objective:**

**To** determine frequencies and clinical features of residual corticosteroid production in patients with AAD.

**Design:**

Two-staged, cross-sectional clinical study in 17 centers (Norway, Sweden, and Germany). Residual glucocorticoid (GC) production was defined as quantifiable serum cortisol and 11-deoxycortisol and residual mineralocorticoid (MC) production as quantifiable serum aldosterone and corticosterone after > 18 hours of medication fasting. Corticosteroids were analyzed by liquid chromatography–tandem mass spectrometry. Clinical variables included frequency of adrenal crises and quality of life. Peak cortisol response was evaluated by a standard 250 µg cosyntropin test.

**Results:**

Fifty-eight (30.2%) of 192 patients had residual GC production, more common in men (n = 33; *P* < 0.002) and in shorter disease duration (median 6 [0-44] vs 13 [0-53] years; *P* < 0.001). Residual MC production was found in 26 (13.5%) patients and associated with shorter disease duration (median 5.5 [0.5-26.0] vs 13 [0-53] years; *P* < 0.004), lower fludrocortisone replacement dosage (median 0.075 [0.050-0.120] vs 0.100 [0.028-0.300] mg; *P* < 0.005), and higher plasma renin concentration (median 179 [22-915] vs 47.5 [0.6-658.0] mU/L; *P* < 0.001). There was no significant association between residual production and frequency of adrenal crises or quality of life. None had a normal cosyntropin response, but peak cortisol strongly correlated with unstimulated cortisol (r = 0.989; *P* < 0.001) and plasma adrenocorticotropic hormone (ACTH; r = –0.487; *P* < 0.001).

**Conclusion:**

In established AAD, one-third of the patients still produce GCs even decades after diagnosis. Residual production is more common in men and in patients with shorter disease duration but is not associated with adrenal crises or quality of life.

Autoimmune Addison disease (AAD) is generally considered to be irreversible, inevitably leading to total destruction of the functional adrenal cortex (1). However, increasing evidence indicates that a subgroup of patients retain some level of corticosteroid production even after many years of disease duration.

In 2011, Smans and Zelissen found quantifiable baseline cortisol levels in 7 of 27 patients with established AAD, measured in a medication fasting state (2). More recently, Vulto et al reported measurable levels of the cortisol precursor, 11-deoxycortisol, in 8 of 20 patients with primary adrenal insufficiency (3). Efforts to exploit residual production therapeutically have demonstrated partial improvement in peak cortisol response to cosyntropin stimulation testing in 7 of 13 patients with newly diagnosed AAD after 12 weeks combined treatment with rituximab and depot tetracosactide (4). In 4 of these patients, stimulated serum cortisol exceeded 100 nmol/L after 72 weeks. At study start, these 4 patients had higher mean stimulated cortisol levels, but did otherwise not differ from the 9 other patients.

Up until now, studies have been performed only in small cohorts, and the clinical relevance of residual production has not yet been addressed. Residual glucocorticoid (GC) production could partly explain observed discrepancies in outcome for patients with AAD. Clinical experience shows great differences in dosage needs for GC replacement therapy, and not all patients require mineralocorticoid (MC) replacement (5). Moreover, 50% of patients with AAD have never experienced an adrenal crisis, and 10% have never required extra GC doses (6). Finally, there are large variations in self-assessed health-related quality of life (HRQoL) in AAD that could potentially be attributed to residual production (7, 8).

Here, we aimed to determine the frequency of residual corticosteroid production in established AAD and to examine the clinical features of residual production.

## Material and Methods

### Participants

We recruited study participants among patients enrolled in the Norwegian Registry of Organ-Specific Autoimmune Diseases, the Swedish Addison Registry, and patients receiving follow-up at the endocrine center “Endokrinologie in Charlottenburg” in Berlin, Germany. Invitation letters were sent to eligible candidates by mail or handed out at regular clinical visits. All included participants had confirmed autoimmune etiology with presence of 21-hydroxylase antibodies, were prescribed GC replacement therapy, and were between 18 and 75 years of age at screening. Exclusion criteria were diabetes mellitus type 1, cancer, severe organ failure, pregnancy, lactation, and current use of medications with known pharmaceutical interactions with adrenocortical hormones (antiepileptics, rifampicin, St John's wart). Any comorbidity had to be stable for at least 3 months before inclusion.

Only patients on hydrocortisone or cortisone acetate replacement therapy were included. Patients previously using dual-release hydrocortisone were switched to cortisone acetate or hydrocortisone at least 1 week prior to blood sampling. Any dehydroepiandrosterone treatment was paused for at least 1 week; alternatively androgen measurements were excluded from statistical analyses. Use of prednisolone or exogenous GCs on indication(s) other than adrenal insufficiency was paused for at least 3 months before blood sampling. Patients using any other antihypertensive medication(s) than alpha blockers or calcium channel blockers, including diuretics, were excluded from analyses on electrolytes, renin, and MC hormones. Patients were instructed to abstain from grapefruit juice and licorice for at least 1 week and caffeinated drinks for at least 24 hours before blood sampling.

### Study design

**Figure 1.**
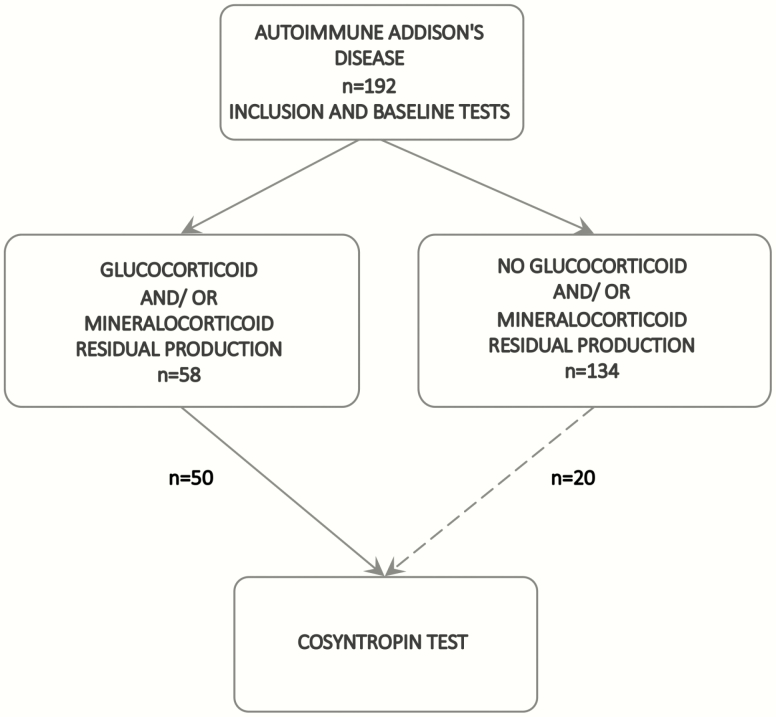
Flow chart of study procedures.

From September 2018 through January 2020 we performed a 2-staged, cross-sectional multicenter clinical study comprising patients with AAD at 17 hospitals in Norway, Sweden, and Germany (Fig. 1). All authors vouch for the accuracy of the data and for the fidelity of the study protocol.

Written informed consent was obtained from all participants before study entry. At stage 1, we registered patient characteristics including age, sex, disease duration, medications, self-reported frequency of adrenal crises and infections, comorbidities, autoimmune polyendocrine syndrome type 2 (APS2), disease-related symptoms, physical health (body mass index [(BMI], blood pressure, and presence of hyperpigmentation), and HRQoL questionnaires. All participants were prescribed hydrocortisone for intramuscular use and instructed to take their replacement medications upon symptoms of precipitating adrenal crisis. Thereafter, patients returned on an agreed morning for medication fasting blood sampling after abstaining from GC and MC intake not later than 2 pm and 8 am the day before, respectively.

At stage 2, patients with quantifiable levels of serum cortisol and 11-deoxycortisol and/or quantifiable levels of serum aldosterone and corticosterone were asked to return for a standard 250 μg cosyntropin stimulation test (Synacthen). Blood samples were collected before (0 minutes) and 30 and 60 minutes after intravenous injection of cosyntropin. Participants with a long commute to the hospital were offered to combine screening and stimulation testing on the same day. At Haukeland University Hospital, we also invited all patients without quantifiable serum cortisol and 11-deoxycortisol to serve as negative controls. Before testing, patients abstained from their steroid replacement therapy in the same manner as described above. A normal response was defined as peak cortisol exceeding 412 or 485 nmol/L after 30 or 60 minutes, respectively (9). The peak response was defined as the highest serum cortisol value recorded at either 30 or 60 minutes.

### Outcomes

The primary endpoint was frequency of residual GC and/ or MC production in patients with AAD. Secondary endpoints included comparison of patients with and without residual GC and/or MC production with regards to patient characteristics including age, sex, disease duration, steroid replacement therapy, peak cortisol in cosyntropin testing, frequency of adrenal crises and infections, physical health (BMI, blood pressure, presence of hyperpigmentation), and HRQoL.

### Laboratory tests

**Figure 2.**
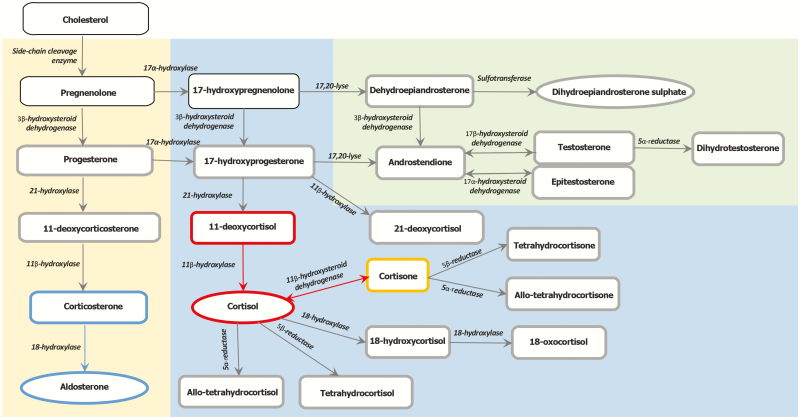
Synthesis of adrenocortical steroids. The 3 main adrenocortical steroids (aldosterone, cortisol, and dihydroepiandrostendione sulphate) are shown in circles, while precursor steroids and metabolites are shown in rectangles. Bold borders mark steroids analyzed in this study. Cortisol and 11-deoxycortisol define residual glucocorticoid production and are marked in red. Aldosterone and corticosterone define residual mineralocorticoid production and are marked in blue. Red and blue arrows mark the enzymatic reactions for activation of cortisol and aldosterone, respectively. Cortisone is both a metabolite and precursor of cortisol and is marked in yellow.

Routine blood tests were analyzed locally: hemoglobin, glycated hemoglobin, thyroid-stimulating hormone, free thyroxine, cobalamin, ferritin, creatinine, sodium, potassium, cholesterol, high-density lipoprotein cholesterol, low-density lipoprotein cholesterol, triglycerides, thyroid peroxidase antibodies, ACTH, and plasma renin concentration (PRC). Levels of ACTH exceeding the upper limit of quantification were plotted as 278 pmol/L. All corticosteroid analyses were performed at Haukeland University Hospital by a liquid chromatography–tandem mass spectroscopy (LC-MS/MS) assay further developed from and expanded on a published method (10), measuring cortisol, 11-deoxycortisol, 21-deoxycortisol, cortisone, 18-oxocortisol, 18-hydroxycortisol, tetrahydrocortisol, allo-tetrahydrocortisol, tetrahydrocortisone, allo-tetrahydrocortisone, aldosterone, corticosterone, 11-deoxycorticosterone, androstendione, testosterone, epitestosterone, dihydrotestosterone, and progesterone (Fig. 2). The lower limit of quantification for each corticosteroid is listed in Table 1.

**Table 1.** Corticosteroids in Patients with and Residual Glucocorticoid Production

			Median (minimum-maximum)		
Corticosteroid	N	LLoQ	GC+	GC–	*P*
18-oxo-cortisol (nmol/L)	192	0.046	0.00 (0.00-0.30)	0.00 (0.00-1.27)	<0.001^a^
18-OH-cortisol (nmol/L)	192	0.046	0.26 (0.00-0.28)	0.00 (0.00-0.20)	<0.001^a^
Aldosterone (pmol/L)^b^	191	8.0	0 (0-220)	0 (0-25)	<0.001^a^
Cortisone (nmol/L)	191	0.914	10.21 (1.63-46.88)	0.00 (0.00-4.16)	<0.001^a^
Cortisol (nmol/L)^c^	192	0.914	57.28 (5.48-507.04)	0.98 (0.00-27.18)	<0.001^a^
DHEAS (nmol/L)	176	22.9	432.69 (25.07-2400.12)	0.00 (0.00-1459.51)	<0.001^a^
21-deoxycortisol (nmol/L)	192	0.023	0.032 (0.00-14.50)	0.00 (0.00-1.05)	<0.001^a^
Corticosterone (nmol/L)	191	0.114	3.50 (0.00-50.84)	0.00 (0.00-2.67)	<0.001^a^
Allo-tetrahydrocortisol (nmol/L)	191	0.114	2.14 (0.00-21.54)	0.00 (0.00-1.56)	<0.001^a^
11-deoxycortisol (nmol/L)	192	0.114	0.60 (0.12-2.87)	0.00 (0.00-0.21)	<0.001^a^
Tetrahydrocortisol (nmol/L)	192	0.343	1.57 (0.00-17.06)	0.00 (0.00-2.84)	<0.001^a^
Allo-tetrahydrocortisone (nmol/L)	192	0.343	0.00 (0.00-1.39)	0.00 (0.00–0.42)	<0.001^a^
Tetrahydrocortisone (nmol/L)	192	0.114	0.95 (0.00–9.82)	0.00 (0.00-0.69)	<0.001^a^
Androstendione (nmol/L)	175	0.023	0.92 (0.00-4.51)	0.440 (0.00-4.04)	<0.001^a^
11-deoxycorticosterone (nmol/L)	191	0.023	0.12 (0.00-0.94)	0.00 (0.00-0.16)	<0.001^a^
Testosterone (nmol/L)	176	0.023	7.74 (0.04-27.39)	0.34 (0.00-30.57)	<0.001^a^
DHEA (nmol/L)	174	0.617	0.71 (0.00-4.33)	0.34 (0.00-1.97)	<0.001^a^
17-hydroxy-progesterone (nmol/L)	192	0.114	2.90 (0.00-49.29)	0.73 (0.00-894.6)	<0.001^a^
Epitestosterone (nmol/L)	176	0.023	0.06 (0.00-0.31)	0.00 (0.00-0.46)	0.008^a^
Dihydrotestosterone (nmol/L)	176	0.206	0.57 (0.00-2.50)	0.00 (0.00-2.61)	0.020
Progesterone (nmol/L)	191	0.114	0.18 (0.00-81.35)	0.00 (0.00-48.27)	<0.001^a^

GC+, residual glucocorticoid production; GC–, no residual glucocorticoid production;

Abbreviations: DHEA, dehydroepiandrosterone; DHEAS, dehydroepiandrosterone sulfate; GC, glucocorticoid; LLoQ, lower limit of quantification.

^a^Statistically significant at 0.01 level.

^b^To convert serum aldosterone values (pmol/L) to ng/dL, divide by 27.7.

^c^To convert serum cortisol values (nmol/L) to μg/dL, divide by 27.6.

### Defining residual corticosteroid production

There is no consensus on the definition of residual corticosteroid production, and no marker of endogenous GC or MC production exists. Here, we defined residual GC production as quantifiable levels of serum cortisol (>0.914 nmol/L) and 11-deoxycortisol (>0.114 nmol/L) and residual MC production as quantifiable levels of serum aldosterone (> 8 pmol/L) and corticosterone (>0.114 nmol/L). All blood samples were obtained in the morning after at least 18 hours without hydrocortisone or cortisone acetate and at least 24 hours without fludrocortisone (FC).

### HRQoL questionnaires

All patients filled out 1 generic (RAND-36) (11) and 1 AAD-specific (AddiQoL) (12) questionnaire assessing HRQoL. RAND-36 is a license free version of the Short Form 36-item (SF-36). It comprises 36 items assessing 8 health concepts: physical functioning, role limitations caused by physical health problems, role limitations caused by emotional problems, social functioning, general mental health, vitality, bodily pain, and general health. Scoring of RAND-36 is a 2-step process. First, precoded numeric values are recorded to a number between 0 and 100 where a higher score represents a better health state. In the second step, items belonging to the same health concept are averaged to create 1 of the 8 total scores (11). AddiQoL has been validated and translated into several languages including Norwegian, Swedish, and German (12). The questionnaire contains 30 items divided into 4 domains: fatigue, emotional well-being, adrenal insufficiency-related symptoms, and miscellaneous (sexuality, sleep, and impact of intercurrent disease). Every item has 6 scoring categories scored as 1, 2, 2, 3, 3, and 4 for positive statements and 4, 3, 3, 2, 2, and 1 for negative statements. A total score is generated by adding the score of individual items, producing a total score ranging from 30 to 120 where a higher score indicates a more favorable HRQoL. A missing individual item score can be replaced by the mean score from the rest of the items in the same subdimension.

### Statistics

We report the primary endpoint as absolute numbers and percentages. Descriptive statistics and secondary endpoints are presented as numbers and percentages for categorical data and as medians and range [minimum to maximum] or as means and standard deviations (± SD) for continuous variables. To compare subgroups, we used independent samples *t* test, Mann-Whitney independent sample U test, and chi-square test, as appropriate. Correlations were explored using the Spearman rank correlation. Binary logistic regression was performed to assess the impact of key patient characteristics on the likelihood of having residual GC or MC production. Nine clinically relevant variables were included: age at diagnosis, sex, disease duration, history of adrenal crisis ever, BMI, hydrocortisone-equivalent dosage (mg cortisone acetate/1.25 = mg hydrocortisone), FC dosage, AddiQoL-30 score, and plasma ACTH (for GC) or PRC (for MC). Preliminary analyses were conducted to ensure no violation of the assumption of multicollinearity. Results are presented as odds ratio (OR) and 95% confidence interval (CI). To reduce the risk of type I error, the alpha value was set to 0.01.

### Ethics

Ethical approval was granted from all participating countries before study start, by the Regional Ethical Committee of South-East Norway (permit no. 2018/751/REK Sør-Øst), of Stockholm, Sweden (permit no. 2018/2247-32), and of Berlin, Germany (permit no. Eth-47/18). The study was registered at clinicaltrials.gov (ClinicalTrials.gov Identifier: NCT03793114) and conducted in agreement with local and international guidelines and regulations, including the Declaration of Helsinki (2013 version) and the principles of good clinical practice (CPMP/ICH/135/95).

## Results

### Stage 1: Frequency and clinical characteristics of residual corticosteroid production

**Figure 3.**
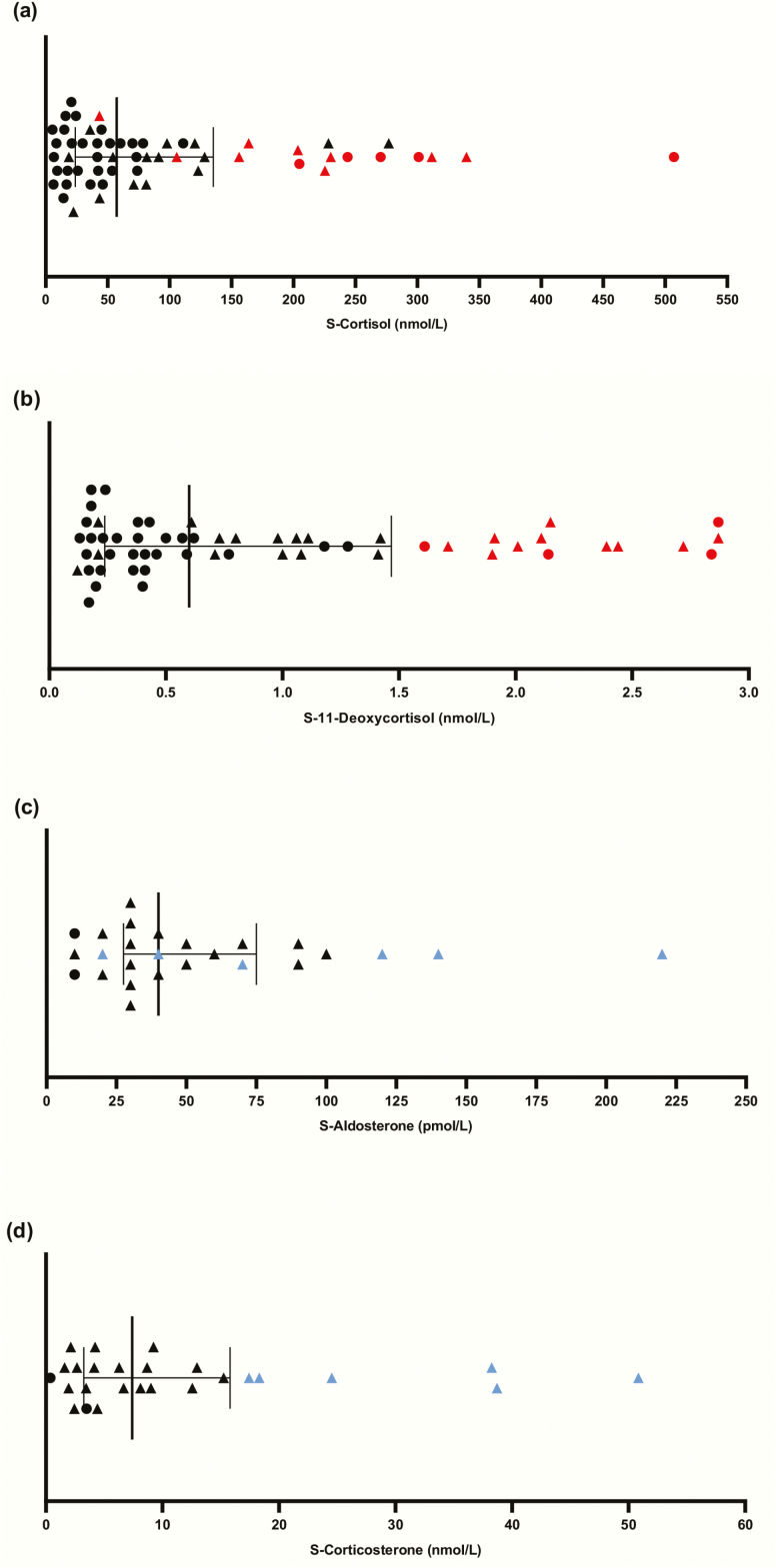
Stage 1: Corticosteroid levels in patients with residual glucocorticoid or mineralocorticoid production. The line marks median corticosteroid values and the whiskers the interquartile range. Triangles mark patients with both glucocorticoid and mineralocorticoid residual production. The patients with the highest quartile of 11-deoxycortisol and corticosterone values are marked in red and blue, respectively. (**A**) Serum cortisol at baseline (n = 58). (**B**) Serum 11-deoxycortisol values at baseline (n = 58). (**C**) Serum aldosterone values at baseline (n = 26). (**D**) Serum corticosterone values at baseline (n = 26).

**Figure 4.**
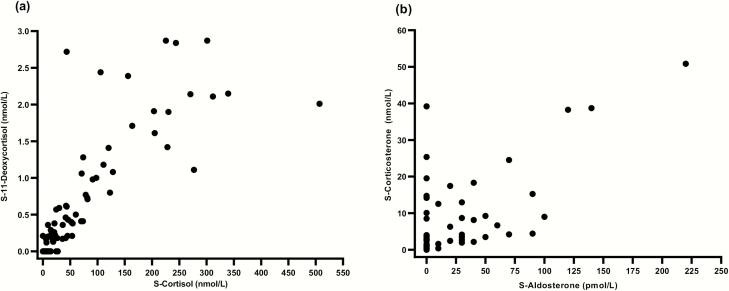
Correlation between corticosteroids. (**A**) Correlation between serum cortisol and 11-deoxycortisol (*P* < 0.001). (**B**) Correlation between serum aldosterone and corticosterone (*P* < 0.001).

Frequency **of residual production.** We included 197 patients with AAD. Five patients declined to proceed to medication fasting blood sampling and were excluded from the study. Baseline characteristics for the remaining 192 patients are presented in Table 2. The medication fast was generally well-tolerated, with only a few individuals reporting increased tiredness and/or headache at blood sampling. Fifty-eight (30.2%) patients had quantifiable levels of serum cortisol and 11-deoxycortisol (Fig. 3A, B), and 26 (13.5%) patients had quantifiable levels of serum aldosterone and corticosterone (Fig. 3C, D). In 24 (12.5%) patients, all 4 hormones were quantifiable. There was a strong positive correlation between serum cortisol and 11-deoxycortisol levels (r = 0.796; P < 0.001) (Fig. 4A), as well as for aldosterone and corticosterone (r = 0.605; P < 0.001) (Fig. 4B).

**Table 2.** Patient characteristics (n = 192)

Characteristics	Number (%) or Median (range) or Mean (±SD)
Female	116 (60.4)
Age (years)	48.3 ± 13.0
Age at diagnosis, years	33.5 (11-64)
Disease duration, years	11 (0-53)
APS 2, n (%)	109 (56.8)
Use of hydrocortisone, n (%)	74 (38.5)
Use of cortisone acetate, n (%)	118 (61.5)
Hydrocortisone equivalent doses, mg/day	20 (7.5-50.0)
Use of fludrocortisone, n (%)	189 (98.4)
Total fludrocortisone dose, mg/day	0.10 (0.03-0.30)
Women using DHEA, n (%)	16 (13.8)
Body mass index, kg/cm^2^	24.4 (16.6-38.3)
Systolic blood pressure, mmHg	120 (84-169)
Diastolic blood pressure, mmHg	76 (50-95)
Hyperpigmentation, n (%)	100 (52.4)

Abbreviations: APS, autoimmune polyendocrine syndrome; DHEA, dehydroepiandrosterone; SD, standard deviation.

**Residual GC production.** Thirty-three (56.9%) of the 58 patients with residual GC production were men (X^2^(1, N = 192) = 9.405; *P* < 0.002). Patients with residual GC production also had significantly shorter disease duration (median 6 [0-44] vs 13 [0-53] years; *P* < 0.001) and higher levels of all adrenal steroids except 18-oxo-cortisol (Table 1). These findings were supported by binary logistic regression, where male sex (OR 5.9; 95% CI, 2.4-14.5; *P *< 0.001) and short disease duration both predicted residual GC production (OR 0.95; 95% CI, 0.91-0.98; *P* < 0.006). As a whole, the regression model explained between 18.5% and 26.3% of the variance in residual GC production status and correctly classified 75.3% of the cases (X^2^(9, N = 182) = 37.308; *P* < 0.001).

The highest recorded serum cortisol value (507 nmol/L) was found in a 68 year-old woman. At time of diagnosis 10 years earlier, she used estrogen replacement therapy. She was admitted due to weight loss, stomach pain, nausea and vomiting and had hyponatremia (124 mmol/L). Although serum cortisol was within normal range, plasma ACTH was elevated at 294 pmol/L, the maximal cortisol peak at cosyntropin test was suboptimal at 407 nmol/L, and the 21-hydroxylase autoantibody index was clearly elevated. Her symptoms were relieved after initiation of replacement therapy with hydrocortisone and FC. In addition, ACTH analyses, cosyntropin tests, and 21-hydroxylase autoantibody assays have been performed at several occasions after diagnosis and remained pathological. The patient reported several adrenal crises since receiving the diagnosis in 2010, including 1 incident last year due to gastrointestinal infection with vomiting and diarrhea.

Residual **MC** production. On group level, patients with MC residual production had shorter disease duration (median 5.5 [0.5-26.0] vs 13 [0-53] years; *P*< 0.004), lower FC replacement dosage (median 0.075 [0.050-0.120] vs 0.100 [0.028-0.300] mg; *P* < 0.005), higher PRC (median 179 [22-915] vs 47.5 [0.6-658.0] mU/L; *P* < 0.001), and higher levels of all but 5 steroids (18-oxo-cortisol, allo-tetrahydrocortisone, testosterone, epitestosterone, dihydrotestosterone; data not shown). For binary logistic regression on residual MC production, only PRC and disease duration significantly contributed to the model. The likelihood of residual MC production decreased with disease duration (OR 0.89; CI 95%, 0.82-0.96; *P*< 0.003) and slightly increased with higher PRC (OR 1.005; CI 95%, 1.002-1.008; *P* < 0.001. In sum, the regression model explained between 18.9% and 35.4% of the variance and correctly classified 90.8% of the cases (X^2^(9, N = 173) = 36.197; *P* < 0.001).

The highest serum aldosterone level recorded (217 pmol/L) was found in a 23-year-old woman. Her plasma renin concentration exceeded the upper limit of detection (>500 mU/L). The patient also presented with a high cortisol (340 nmol/L) and did not use oral contraceptive pills or estrogen. Of note, the patient had experienced adrenal crisis twice since receiving the diagnosis in 2013 and suffers concomitant hypothyroidism, celiac disease, vitamin B12 deficiency, and previously Graves disease. At time of diagnosis, she fulfilled the diagnostic criteria for AD, with morning cortisol in the lower reference range, elevated ACTH level, and clearly elevated index of 21-hydroxylase autoantibodies.

**Combined residual GC and MC production.** Twenty-four patients had quantifiable levels of cortisol, 11-deoxycortisol, aldosterone, and corticosterone. They had significantly shorter disease duration (median 5.5 [0.5-26.0] vs 13.5 [0.0-53.0] years; *P* < 0.002), higher PRC (median 152 [22-915] vs 46 [1-658] mU/L; *P* < 0.001), and higher levels of all but 3 steroids (testosterone, epitestosterone, dihydrotestosterone; data not shown) compared with patients with no residual production. Individual patient data are presented in Table 3.

**Table 3.** Characteristics of the 24 patients with combined glucocorticoid and mineralocorticoid residual production

	1	2	3	4	5	6	7	8	9	10	11	12	13	14	15	16	17	18	19	20	21	22	23	24
**S-F, nM**	340	311	277	230	228	225	204	164	156	128	123	120	106	98	91	82	81	71	54	43	43	36	22	19
**S-S, nM**	2.2	2.1	1.1	1.9	1.4	2.9	1.9	1.7	2.4	1.1	0.8	1.4	2.4	1.0	1.0	0.7	0.7	1.1	0.2	0.6	2.7	0.2	0.2	0.1
**S-Aldo, pM**	217	69	135	121	56	25	14	15	86	104	39	39	15	92	53	28	29	51	66	31	24	38	10	33
**S-CCN, nM**	51	25	39	38	7	13	13	17	15	9	18	8	6	4	9	9	3	4	4	4	2	2	2	2
**Sex, M or F**	F	M	F	F	M	F	M	F	F	F	M	F	F	F	F	F	M	F	F	M	F	M	M	F
**Age, years**	23	63	32	43	62	51	18	47	40	59	30	53	55	51	49	53	18	45	66	26	23	38	36	42
**DD, years**	5	6	1	5	26	9	5	7	0.5	6	4	24	9	20	5	1	2	7	2	2	1	21	7	12
**AC, yes or no**	yes	yes	no	yes	yes	no	yes	yes	yes	no	yes	yes	no	no	yes	no	yes	no	yes	yes	no	no	yes	yes
**HCeq., mg**	20	25	20	20	20	10	28	30	30	20	25	20	15	10	17.5	20	30	20	20	40	30	30	30	25
**BMI, kg/m** ^**2**^	18.1	25.6	24.4	23.0	26.1	23.3	27.8	29	34.3	37.1	28.7	27.1	26.0	25.9	27.8	29.4	20.8	22	21.0	21.2	22.0	26.5	22.3	18.8
**AddiQoL score**	66	89	72	102	94	95	90	105	73	117	86	80	93	95	80	96	102	101	93	99	83	91	*	91
**P-ACTH, pmol/L**	63	32	26	70	34	68	39	210	62	237	82	67	120	134	88	259	224	225	31	278	125	43	278	175
**PRC, mU/L**	500	187	146	302	48	308	76	325	122	124	179	22	*	465	61	337	77	107	22	152	350	214	81	915

Abbreviations: AAD, autoimmune Addison disease; AC, adrenal crisis ever; AddiQoL, AAD-specific questionnaire; BMI, body mass index; DD, disease duration; F, female; HCeq, hydrocortisone-equivalent dose; M, male; P-ACTH, plasma adrenocorticotropic hormone; PRC, plasma renin concentration; S-Aldo, serum aldosterone; S-CCN, serum corticosterone S-F, serum cortisol, S-S, serum 11-deoxycortisol.

* Not obtained.

Residual production and clinical characteristics. On group level, all routine laboratory values were within the reference intervals (Table 4). Patients with residual GC and/or MC production did not differ significantly from those without residual production regarding frequency of adrenal crises, number of infections the previous year, APS2, disease-related symptoms, hydrocortisone equivalent dosage, physical health, or HRQoL scores (AddiQoL and RAND-36) (Table 4).

**Table 4.** Differences in patient characteristics between patients with and without residual glucocorticoid production and in patients with combined glucocorticoid and mineralocorticoid production compared with patients with no residual production.

	N (%) or Median (minimum, maximum) or Mean (±SD)					
Variable	GC+	GC–	*P*	GC+, MC+	GC–, MC–	*P*
No. females (%)	25 (43.1)	91 (67.9)	0.001^a^	16 (66.7)	89 (67.4)	1.000
Age, years	46.2 ± 14.8	49.2 ± 12.3	0.142	42.6 ± 14.4	49.3 ± 12.3	0.020
DD, years	6 (0-44)	13 (0-53)	0.001^a^	5.5 (0.5-26)	13.5 (0.0-53.0)	0.002^a^
Age at diagnosis, years	36 (12-64)	31.5 (11-63)	0.202	33.5 (13-64)	31 (11-63)	0.673
Adrenal crisis ever, n (%)	38 (65.6)	98 (73.7)	0.252	15 (62.5)	96 (73.3)	0.406
Adrenal crisis at diagnosis, no (%)	34 (58.6)	79 (59.4)	0.920	13 (54.2)	78 (59.5)	0.790
Adrenal crisis last year, n (%)	11 (19.0)	19 (14.3)	0.414	5 (20.8)	19 (14.5)	0.630
Infectious illness last year, n (%)	22 (37.9)	49 (37.1)	0.915	7 (29.2)	49 (37.7)	5.710
CVD, no. (%)	0 (0)	2 (1.5)	0.350	0 (0)	2 (1.5)	1.000
Osteoporosis, n (%)	4 (6.9)	12 (9.0)	0.626	0 (0)	12 (9.2)	0.259
APS2, no. (%)	29 (50)	80 (59.7)	0.277	14 (58.3)	78 (59.1)	0.945
Salt cravings, n (%)	14 (24.1)	33 (24.6)	0.942	8 (33.3)	32 (24.2)	0.494
Orthostatic hypotension, n (%)	14 (24.1)	18 (13.4)	0.068	8 (33.3)	18 (13.6)	0.037
Fatigue, n (%)	22 (37.9)	54 (40.3)	0.758	7 (29.2)	54 (40.5)	0.391
Loss of appetite, n (%)	6 (10.3)	6 (4.5)	0.123	2 (8.3)	6 (4.5)	0.786
GI-symptoms, n (%)	14 (24.1)	25 (18.7)	0.386	5 (20.8)	25 (18.9)	1.000
Muscle/ joint pain, n (%)	17 (29.3)	33 (24.6)	0.497	4 (16.7)	33 (25.0)	0.534
Sleeping disturbances, n (%)	20 (34.5)	36 (26.9)	0.286	11 (45.8)	36 (27.3)	0.114
Nausea, n (%)	4 (6.9)	10 (7.5)	0.890	4 (16.7)	10 (7.6)	0.296
BMI, kg/m^2^	25.1 (18.1-37.1)	24.1 (16.6-38.3)	0.257	25.8 (18.1-37.1)	24.1 (16.6-38.3)	0.512
SBP, mmHg	120.5 (90-150)	120 (84-169)	0.019	120 (90-150)	120 (84-169)	0.356
DPB, mmHg	76 (55-93)	76 (50-95)	0.537	75 (55-90)	76 (50-95)	0.737
Hyperpigmentation, n (%)	29 (50.0)	71 (53.4)	0.667	11 (45.8)	70 (53.4)	0.643
HCeq, mg/day	20 (10-50)	20 (7.4-40)	0.875	20 (10-40)	20 (7.5-40)	0.375
HCeq, mg/kg/day	0.31 (0.14-0.58)	0.32 (0.12-0.78)	0.179	0.31 (0.14-0.55)	0.32 (0.03-0.3)	0..484
HCeq, mg/m^2^/day	7.4 (3.3-13.3)	7.7 (2.8-15.4)	0.179	7.9 (3.3-12.0)	7.8 (2.8-15.4)	0.839
FC, mg/ day	0.10 (0.05-0.20)	0.10 (0.03-0.30)	0.156	0.10 (0.05-0.12)	0.10 (0.03-0.30)	0.014
RAND-36 PF	95 (55-100)	95 (25-100)	0.710	95 (80-100)	95 (35-100)	0.395
RAND-36 RP	100 (0-100)	100 (0-100)	0.444	100 (0-100)	100 (0-100)	0.087
RAND-36 BP	74 (22-100)	84 (12-100)	0.835	84 (22-100)	83 (12-100)	0.363
RAND-36 GH	67 (17-100)	67 (5-95)	0.879	67 (20-97)	67 (10-100)	0.718
RAND-36 VT	65 (5-100)	60 (5-95)	0.407	65 (5-100)	60 (5-95)	0.198
RAND-36 SF	87.5 (25-100)	87.5 (12.5-100)	0.991	87.5 (25-100)	87.5 (12.5-100)	0.653
RAND-36 RE	100 (0-100)	100 (0-100)	0.374	100 (0-100)	100 (0-100)	0.605
RAND-36 MH	80 (36-100)	84 (44-100)	0.534	76 (36-100)	84 (55-100)	0.156
AddiQol-30	90.9 ± 12.7	89.6 ± 10.3	0.476	91 ± 11.7	89.5 ± 10.3	0.5230
Hb, g/dL	14.5 ± 1.2	13.8 ± 1.1	0.001^a^	14.6 ± 1.2	13.8 ± 1.1	0.004^a^
HbA_1C_, mmol/mol	35 (28-53)	35 (24-43)	0.657	35 (28-50)	35 (24-43)	0.806
S-TSH, mIE/L	2.6 (0.05-12.7)	2.55 (0.01-13.2)	0.977	1.8 (0.06-7.0)	2.6 (0.01-13.2)	0.140
S-fT_4_, pmol/L	15.0 (10.6-23.0)	15.0 (10.6-25.0)	0.983	16.2 (11-23)	15.0 (10.6-25.0)	0.262
S-cobalamin, pmol/L	368 (174-753)	372 (140-1476)	0.964	379 (210-605)	372 (140-1476)	0.490
S-ferritin µg/L	116 (15-446)	101 (6-621)	0.394	91 (15-297)	102 (6-621)	0.416
S-creatinine, µmol/L	77 (60-150)	73 (39-116)	0.006^a^	75 (60-96)	73 (39-116)	0.405
S-sodium, mmol/L	139 (131-145)	140 (131-148)	0.237	138 (136-142)	140 (131-148)	0.005^a^
S-potassium, mmol/L	4.1 (3.5-4.9)	3.9 (3.0-5.1)	0.008^a^	4.2 (3.5-4.6)	3.9 (3.0-5.1)	0.036
S-cholesterol, mmol/L	5.1 ± 1.0	5.1 ± 0.9	0.630	5.1 ± 1.2	5.1 ± 1.0	0.827
S-HDL-C, mmol/L	1.4 (0.1-2.2)	1.7 (0.6-2.7)	0.001^a^	1.5 (1.1-2.2)	1.7 (0.6-2.7)	0.224
S-LDL-C, mmol/L	3.2 ± 1.0	3.1 ± 0.8	0.406	3.3 ± 1.2	3.1 ± 0.8	0.292
S-triglycerides, mmol/L	1.3 (0.4-5.8)	1.3 (0.1-9.7)	0.609	1.2 (0.5-3.2)	1.3 (0.1-9.7)	0.702
PRC, mU/L	75.7 (0.7-915.0)	49.0 (0.6-658.0)	0.055	152 (22-915)	46 (1-658)	<0.001^a^
P-ACTH, pmol/L	123 (26-278)	147 (1-278)	0.123	85 (26-278)	147 (1-278)	0.087

Abbreviations: APS2, autoimmune polyendocrine syndrome type 2; BMI, body mass index; BP, bodily pain; CVD, cardiovascular disease; DBP, diastolic blood pressure; DD; disease duration; FC, fludrocortisone dosage; fT_4_, free thyroxine; GC+, residual glucocorticoid production; GC–, no residual glucocorticoid production; GH, general health; GI, gastrointestinal; Hb, hemoglobin; HbA_1c_, glycated hemoglobin; HCeq, hydrocortisone equivalent dosage; HDL-C, high-density lipoprotein cholesterol; LDL-C, low-density lipoprotein cholesterol; MC+, residual mineralocorticoid production; MC–, no residual mineralocorticoid production; MH, general mental health; P-ACTH, plasma adrenocorticotropic hormone; PF, physical functioning; PRC, plasma renin concentration; RAND, health survey; RE, role limitations caused by emotional problem; RP, role limitations caused by physical health problems; S-, serum; SBP, systolic blood pressure; SD, standard deviation; SF, social functioning; TSH, thyroid-stimulating hormone; VT, vitality.

^a^Statistically significant at 0.01 level.

### Stage 2: Cosyntropin test

**Figure 5.**
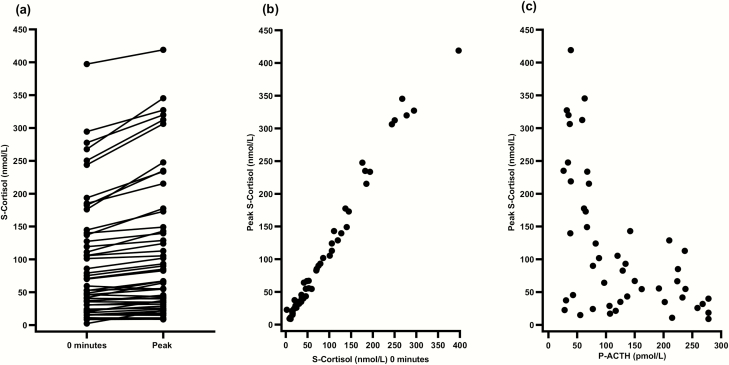
Cosyntropin testing. (**A**) Change in serum cortisol before (0 minutes) intravenous 250 μg cosyntropin to peak serum cortisol after 30 or 60 minutes. (**B**) Correlation between serum cortisol before (0 minutes) intravenous 250 μg cosyntropin and peak serum cortisol at 30 or 60 minutes (*P* < 0.001). (**C**) Correlation between plasma ACTH before (0 minutes) intravenous 250 μg cosyntropin and peak serum cortisol 30 or 60 minutes (*P* < 0.001). ACTH, adrenocorticotropic hormone.

In total, 55 patients with residual GC production underwent the cosyntropin test. Three patients with quantifiable cortisol and 11-deoxycortisol at baseline declined. The screening results of residual GC production were verified in all but 5 patients. These patients were excluded from statistical analyses on cosyntropin test results. The remaining 50 patients reached a median peak cortisol of 75 [9-419] nmol/L (Fig. 5A), confirming the diagnosis of adrenal insufficiency. Higher serum cortisol levels at 0 minutes and lower plasma ACTH levels strongly correlated with peak cortisol (r = 0.989; *P* < 0.001, and r = –0.487; *P* < 0.001, respectively) (Figs. 5B and 5C).

The cosyntropin test was also performed in 2 patients with isolated residual MC production at screening, but upon testing aldosterone, it was only quantifiable for 1 of them. For this patient, aldosterone levels remained unchanged at 40 pmol/L throughout the test.

Twenty patients without quantifiable levels of cortisol and 11-deoxycortisol and/or aldosterone and corticosterone at stage 1 were included as controls. At cosyntropin testing, serum cortisol was barely quantifiable in 10 of the controls but remained unquantifiable in the other 10 controls. Two controls also had barely quantifiable levels of serum corticosterone, but none had quantifiable levels of serum 11-deoxycortisol or aldosterone.

## Discussion

We found residual GC production in one-third of patients with established AAD, more common in men than in women. Patients with residual production had overall shorter disease duration, but several had a history of AAD lasting for decades. More than 1 of 7 patients had residual MC production. These were characterized by shorter disease duration, lower FC dosage, and higher plasma renin concentrations compared with those without residual MC production. No significant associations were found between residual corticosteroid production and a number of clinical parameters. To date, this is the largest study on residual production in AAD, conducted on a representative study cohort from 17 centers in 3 countries. We are confident that the diagnosis of AAD is correct in all included patients as we required documented presence of 21-hydroxylase antibodies and chronic need for GC replacement therapy for inclusion.

There is no established definition of residual corticosteroid production. LC-MS/MS enables measurement of minute quantities of cortisol and aldosterone; however, the clinical effect of very low cortisol and aldosterone concentrations is uncertain. We believe that merely evaluating serum cortisol levels would result in a falsely high prevalence of residual GC production, as up to half of the bioavailable cortisol stems from cortisone regenerated by 11-β-hydroxysteroid dehydrogenase type 1 (13). In addition, it is important to discriminate between endogenous and exogenous cortisol in these patients who use GC replacement therapy. This could in part be avoided by having patients abstain from GC replacement therapy for a longer period of time but would put them at risk of developing an adrenal crisis. Concerning residual MC production, we are not aware of any bidirectional pathways in aldosterone metabolism. Furthermore, FC is a synthetic MC and does not interfere with aldosterone measurements on LC-MS/MS (14). In the present study, patients were asked to abstain from GC and MC replacement therapy for at least 18 and 24 hours, respectively, before sample collection. To further ensure that the measured hormones indeed represented de novo synthesis of corticosteroids, we chose to include precursors for the definitions of residual GC and MC production. Importantly, the enzymes involved in conversion of the precursors to the active substances are considered unidirectional (15), precluding any synthesis of precursors from cortisol or aldosterone. This was well illustrated by 1 of the study participants who had a serum cortisol level of 797 nmol/L but no quantifiable 11-deoxycortisol. Later, it become known that she had taken her morning dose of cortisone acetate before the blood sampling but had forgotten to mention it. The patient was therefore excluded. In patients with residual production, we found that median and range values of 11-deoxycortisol and corticosterone corresponded with values found in healthy controls (16), suggesting that these are suitable as biomarkers of residual production.

We were surprised to detect a clear overweight of men with residual GC production, despite women constituting the majority of our study cohort. This may be due to sex-related disparities in immunology as well as susceptibility to autoimmune disease (17). It has been suggested that inherent sex differences in adrenal gland tissue renewal could be involved (18). Indeed, in mice, the turnover rate for adrenocortical tissue is 3 times higher in females compared with males, and capsular stem cells only contribute to tissue renewal in females, not in males (18). Whether these findings are relevant for humans is not known, and highlights the need for future studies to explore the impact of sex on the trajectory of autoimmune adrenalitis.

As expected, the patients with GC and/or MC residual production had shorter median disease duration. However, there was a wide range in disease duration among the patients with residual production, extending up to 26 years for MC and 44 years for GC residual production, arguing against the common assumption that AAD inevitably leads to loss of all adrenal corticosteroid production. Concurrently, it raises questions of how and why the intensity and extent of the autoimmune attack seem to differ between individuals.

Regarding steroid replacement therapy, we found significantly lower dosages of FC in patients with residual MC production. This could, of course, be due to lower replacement needs. As these participants also had higher levels of plasma renin concentration, one could speculate if greater renin exposure via an activated renin-angiotensin-aldosterone system may stimulate MC production in remnants of the zona glomerulosa. We did not find any association between residual corticosteroid production and hydrocortisone-equivalent dosages. This might be masked by the fact that GC receptor polymorphisms influence the GC replacement dose (19). In addition, there is currently no available biomarker to guide optimization of GC replacement treatment. When evaluating FC dosages, the physician is aided by the patient’s blood pressure, electrolyte levels, and plasma renin concentration (20). For GC therapy, however, surveillance relies upon more vague clinical signs and the patient’s subjective health status (21). Therefore, we cannot rule out that patients with residual GC production receive unnecessarily high GC dosages. If true, residual production could put patients at risk of deleterious health effects due to GC excess, including cardiovascular disease (22), infections (23), and premature death (24). Whether residual production enables safe dose reductions should be explored in further studies.

Of note, we found no differences in frequency of adrenal crises, infections, APS2, disease-related symptoms, physical health, or HRQoL in patients with and without residual production of adrenal corticosteroids. An obvious explanation is, of course, that no such links exist. Yet, as with any exploratory study, we must acknowledge that our chosen methods may not have been ideal for evaluating the clinical significance of residual GC and MC production. Furthermore, quantifiable levels of adrenal corticosteroids may not represent clinically significant values. Inaccuracies due to recall bias must also be considered, especially for the frequencies of adrenal crises and infections that were self-reported by the patients.

In line with previous studies, none of the patients in the current study had a normal response to the cosyntropin test (2, 4, 25, 26). Still, patients with higher cortisol levels before injection of cosyntropin reached significantly higher peak cortisol, suggesting a greater stimulatory potential. Indeed, in attempts to regenerate adrenocortical function in AAD by rituximab and/or tetracosactide, lasting recovery has only been reported in 2 patients with cosyntropin-stimulated peak cortisol of 219 and 235 nmol/L before treatment initiation (4, 25, 26).

Unfortunately, our study design did not allow us to answer the compelling questions on the nature and origin of residual production in AAD. In order to investigate possible heterogeneity in disease development and adrenal plasticity, we call for a prospective study including newly diagnosed individuals to be assessed at baseline and followed annually. Such a study could ascertain whether certain AAD subpopulations are more resistant to immune-mediated destruction, perhaps by harboring other human leukocyte antigen genotypes than patients without residual production, or if the intensity of autoimmune destruction may vary over time allowing regeneration of steroid-producing cells.

In our opinion, remnants of functional adrenocortical tissue are the most probable origin of residual production. We suggest 2 possible mechanisms: Either areas in the adrenal cortex have been spared from autoimmune attack or adrenocortical cells could be replenished by differentiation of subcapsular stem cells (27). Both are in line with observations in autoimmune type 1 diabetes where pancreatic infiltration of immune cells is not always uniform but may be patchy and leave subsets of pancreatic islets unaffected (28). Indeed, recent reports suggest that residual beta cell capacity may be present in one-third of patients with longstanding type 1 diabetes (28).

An alternative explanation is extra-adrenal production. The observed male preponderance in residual GC production opens for a tantalizing link to hormone-producing testicular adrenal rest tumors (TARTs), as seen in approximately 40% of men with congenital adrenal hyperplasia (29). However, a recent ultrasonographic screening of 14 men with Addison disease could not detect any cases of TART (30). Moreover, if TARTs indeed were the true sources of residual production, there would still be the question on how cortisol-producing cells evade the autoimmune attack, as the Leydig cells are located outside the blood-testis barrier (31). In conclusion, one-third of patients with autoimmune Addison disease still produce GCs and MCs even years after the diagnosis, more commonly observed in men in our cohort. These findings challenge our current understanding of the natural course of the disease.

## Abbreviations

AAD: autoimmune Addison disease
ACTH: adrenocorticotropic hormone
AddiQoL: AD-specific QoL questionnaire
APS2: autoimmune polyendocrine syndrome type 2
BMI: body mass index
CI: confidence interval
FC: fludrocortisone
GC: glucocorticoid
HRQoL: health-related quality of life
LC: -
MS/MS: liquid chromatography–tandem mass spectrometry
MC: mineralocorticoid
OR: odds ratio
PRC: plasma renin concentration
RAND: HRQoL survey
SD: standard deviation
TART: testicular adrenal rest tumor.

## Additional Information

***Disclosure Statement***: The authors have nothing to disclose.

***Data Availability:*** The datasets generated during and/or analyzed during the current study are not publicly available but are available from the corresponding author on reasonable request.
